# Impact of lockdown on football players’ injuries

**DOI:** 10.1093/eurpub/ckac130.234

**Published:** 2022-10-25

**Authors:** V Falcone, D Romani, N Nante, F Cocchi, G Messina

**Affiliations:** 1 Post Graduate School of Public Health, University of Siena, Siena, Italy; 2 UOC Hygiene and Public Health-Sud Area, Azienda USL Toscana Sud-Est, Grosseto, Italy; 3 Department of Molecular and Developmental Medicine, University of Siena, Siena, Italy; 4 Training and Skills Unit, ASL 1 Roma, Rome, Italy

## Abstract

**Background:**

The global outbreak of COVID-19 has resulted in the closure of stadiums and the interruption of Serie A for three months. Many studies have evaluated the effects of COVID on population health, but few have evaluated the effects of containment measures on the health of football players. With this study, we evaluated the impact of this break on Serie A football players.

**Methods:**

This cross-sectional study was conducted considering a timespan of three Serie A seasons (2018-19; 2019-20; 2020-21). The information was obtained from the German website Transfermarkt. All the players who had played at least one match during each of the Serie A season were identified. For each of the players, data concerning the number of days lost due to injury, both before and after the stop in the championship due to Covid, were collected. Statistical analysis was performed using Stata 17 Software.

**Results:**

According to the selection criteria, 264 players were selected. This group was subsequently skimmed to 256 players after eliminating all players who did not suffer physical injuries over the timespan considered (non-purely orthopedic surgery; COVID; Intestinal problems; Infections). 256 players were analysed, 228 had skipped at least one day for pre-lockdown due to physical injury (median=37,5), while 227 missed a day for post-lockdown (median=27). Wilcoxon signed-rank test between days lost due to injuries before and after lockdown highlighted significant differences (p < 0.05).

**Conclusions:**

Comparing pre-lockdown and post-lockdown periods, we noticed that there were fewer days skipped due to physical injury post lockdown. Statistical evidence suggests that the same players were more susceptible to suffer physical injuries in the pre-lockdown period. This is probably because some players have worked with home coaching by spending more time in the gym and less time on the field. Also tapis roulant and cyclettes were often delivered to football players’ homes.

**Key messages:**

- In this phase of coexistence with COVID-19 it is of fundamental importance to understand the impact that public health measures have on the population.
- Although, with vaccination, there has been a reduction in the number of cases, it is critical to strengthen preparedness against all possible epidemic scenarios.

